# An evaluation of the antidiabetic effects of *Elaeocarpus ganitrus* in experimental animals

**DOI:** 10.4103/0253-7613.75671

**Published:** 2011-02

**Authors:** Amolkumar K. Hule, Abhishek S. Shah, Manoj N. Gambhire, Archana R. Juvekar

**Affiliations:** Department of Pharmaceutical Sciences and Technology, University Institute of Chemical Technology (UICT), Mumbai University, Matunga, Mumbai – 400 019, India

**Keywords:** Antihyperglycemic, diabetes, *Elaeocarpus ganitrus*, streptozotocin

## Abstract

**Objective:**

To evaluate the antidiabetic effects of the aqueous extract of *Elaeocarpus ganitrus* (EAG) in experimental animals.

**Materials and Methods:**

The hypoglycemic activity of the EGA was evaluated in normoglycemic rats by single dose at three graded dose levels, viz. 250, 500 and 1000 mg/kg of body weight. Antihyperglycemic activity of the extract was also evaluated at the same dose levels in streptozotocin (STZ) (60 mg/kg, i.p.)-induced diabetic rats during a 30-day treatment period. Metformin (500 mg/kg) was used as the reference drug. Fasting blood glucose and lipid parameters, viz. triglycerides, total cholesterol, high-density lipoprotein and low-density lipoprotein levels were measured. Acute oral toxicity of the EGA extract was carried out in Swiss albino mice.

**Results:**

In normoglycemic rats, EGA showed a significant (*P* < 0.01) hypoglycemic effect at 2 h. In STZ-induced diabetic rats, the EGA treatment significantly (*P* < 0.05) decreased the blood glucose level in a dose-dependent manner during the 30 days of treatment period. EGA modulated lipid profile changes in STZ-diabetic rats in a dose-dependant manner. In the acute oral toxicity study, EGA showed no mortality till the 5 g/kg dose in mice.

**Conclusion:**

The present investigation shows that EAG seeds has potential antidiabetic effects.

*Elaeocarpus ganitrus* Roxb. (Elaeocarpaceae) is commonly known as Rudraksha in India. Previous studies have shown that *E. ganitrus* possesses sedative, hypnotic, tranquillizing, anticonvulsive, antiepileptic and antihypertensive activities.[1] *E. ganitrus* yielded quercetin, gallic and ellagic acids, (-) elaeocarpine, (-) iso-elaeocarpine and rudrakine.[1]

*E. grandiflorus*, a member of the Elaeocarpaceae family, possesses antidiabetic activity.[2] The water extract of *E. ganitrus* has been traditionally used to treat diabetic patients without scientific data. The objective of the present study was to evaluate the hypoglycemic and antihyperglycemic potential of the aqueous extract of *E. ganitrus* (EGA) in rats.

## Materials and Methods

### 

#### Chemicals

Streptozotocin (STZ) was obtained from Sigma (Sigma-Aldrich Co., USA). All other chemicals used were of analytical grade. Glibenclamide (Daonil; Aventis Pharma) and metformin (Emfor; Stanmed) were obtained from a local dealer. Diagnostic kits for glucose, cholesterol and triglyceride determination were obtained from Merck Specialties Pvt. Ltd., Mumbai, India.

#### Plant material

Authenticated seeds of *E. ganitrus* Roxb were obtained as a gift sample from Rudraksha Research and Testing Laboratory (RRTL), Mumbai, India. The seeds were coarse powdered in a cutter and grinding mill. Powdered seeds of *E. ganitrus* (4 kg) were extracted twice with distilled water (20 L) by stirring overnight and then centrifuged at room temperature. The supernatant was collected and evaporated to dryness at 50°C under pressure in a rotary evaporator. The yield of extract (EGA) was about 2.55% w/w, and this was stored in a desiccator.

#### Animals

Adult male Wistar rats and Swiss albino mice were obtained from Haffkine Bio-Pharmaceuticals Ltd., Mumbai, India. The animals were acclimatized for 10 days before being used for the experiments. They were housed in a room with controlled temperature (23 ± 2C) and a 12-h light/12-h dark cycle. The animals were maintained on a standard dry pellet diet (Amrut Brand, Sangli, India) and water *ad libitum*. The experimental protocol was approved by the Institutional Animal Ethics Committee and was executed according to the guidelines of Committee for the Purpose of Control and Supervision on Experiments on Animals (CPCSEA), India.

#### Phytochemical screening

Freshly prepared EGA extract was tested for the presence of alkaloids, steroid and/or triterpenoids and their glycosides, tannins, flavonoids and their glycosides, carbohydrates and cardiac glycosides using the standard procedure.[3]

#### Acute oral toxicity study

An acute oral toxicity study of EGA extracts for the determination of lethal dose (LD_50_) was carried out in mice by administering different doses according to the method described by Ghosh *et al*.[4] It was observed that the extract was nontoxic up to the dose of 5.0 g/kg body weight and was used in different doses for further studies.[4,5]

#### Effect of EGA in normal rats

The study was carried out to test the effect of *E. ganitrus* on the blood sugar levels in normal rats. The rats weighing 200-240 g were divided into five groups of six animals each. The animals were fasted overnight before the experiment but were allowed free access to water. Group I was treated with vehicle (0.5% sodium carboxy methyl cellulose – NaCMC) and served as normal control. Groups II, III and IV were treated with EGA orally at doses of 250, 500 and 1000 mg/kg body weight, respectively. Group V was administered glibenclamide (10 mg/kg).[6] Blood glucose levels (BGLs) were determined using a commercial diagnostic kit (Merck Specialties Ltd.) at different time intervals, viz. 0 (before drug administration) and 1, 2 and 4 h after drug administration.[7]

#### Effect of EGS on STZ-induced diabetic rats

Diabetes was induced in Wistar rats as per the procedure of Pushparaj.[8] Albino Wistar rats weighing 200-250 g were fasted overnight for 12 h and were injected intraperitoneal (i.p.) freshly prepared STZ (Sigma-Aldrich Co.) dissolved in 10 mM citrate buffer (pH 4.5) at a dose of 60 mg/kg body weight. Hyperglycemia was confirmed by the elevated fasting blood glucose (FBG) levels determined at 72 h and day 7. Rats with FBG between 250 and 350 mg/dl were selected for the study. A mortality of 12% was observed with STZ (60 mg/kg) during the induction of diabetes.

#### Experimental design

Nondiabetic rats (n = 6) treated with vehicle (0.5% NaCMC) were considered as normal control; Group I. Diabetic rats (n = 30) were randomly divided into five groups of six rats each. Group II was treated with vehicle (0.5% NaCMC) and served as the diabetic control. Group III, IV and V animals were treated with oral EGA at dose levels of 250, 500 and 1000 mg/kg, respectively. Group VI was treated with metformin (500 mg/kg). All the animals were treated orally daily for 30 days. FBG levels were measured on day 0, i.e. just prior to the initiation of any treatment, on day 16 and 24 h after the last dose of treatment using commercial diagnostic kits. Serum was separated from the blood samples collected through the retroorbital venous plexus under ether anesthesia 24 h after the last dose. The serum lipid profile, including triglycerides, total cholesterol, high-density lipoprotein (HDL) and low-density lipoprotein (LDL) were determined on these serum samples using commercial diagnostic kits[9,10] (Merck Specialties Pvt. Ltd.).

#### Statistical analysis

All values were expressed as mean ± standard error of mean (SEM). The data obtained were subjected to statistical analysis using one-way ANOVA followed by Dunnett’s multiple comparisons test for the control and multiple test groups. *P* <0.05 was considered to be statistically significant.

## Results

### 

#### Phytochemical screening

The phytochemical studies of EAG revealed the presence of alkaloids, glycosides, steroids and flavonoids.

#### Acute oral toxicity study

Acute oral toxicity observed that EGA was nontoxic and caused no mortality up to 5 g/kg orally in mice. Therefore, the LD_50_ value of EGA is >5 g/kg of body weight.

#### Effect of EGA in normal rats

The EGA extract at the dose levels 500 and 1000 mg/kg of body weight in normal nondiabetic fasted rats showed a significant decrease (*P* < 0.01) in the BGL at 2 h as compared with the control group [Table 1]. The maximum reduction was 28.77% at 1000 mg/kg at 2 h, while glibenclamide reduced the same to 31.28% at 2 h as compared with baseline [Table 1].

**Table 1:** Effect of the aq. extract of *Elaeocarpus ganitrus* on blood glucose levels in normal rats

*Group*	*Treatment*	*Blood glucose (mg/dl)*			
		*0 h*	*1 h*	*2 h*	*4 h*
Control	0.5% NaCMC	92.33 ± 4.8	85.42 ± 2.47	81.27 ± 2.42	77.54 ± 2.28
EGA	250 mg/kg	90.66 ± 4.36	74.88 ± 2.98	65.88 ± 3.13**	69.06 ± 2.38
EGA	500 mg/kg	88.63 ± 5.61	70.65 ± 4.22**	59.38 ± 3.41**	65.6 ± 2.89
EGA	1000 mg/kg	87.65 ± 3.89	65.16 ± 2.55**	57.89 ± 2.64**	62.89 ± 3.63*
Glibenclamide	10 mg/kg	94.02 ± 3.01	62.95 ± 3.12**	55.85 ± 1.88**	61.66 ± 4.54**

Values are expressed as mean ± SEM; *n* = 6;

* *P* < 0.05;

** *P* < 0.01 vs. control group at the respective hour; one-way ANOVA followed by Dunnett’s multiple comparisons test; EGA, aqueous extract of *Elaeocarpus ganitrus*

#### Effect of EGA on STZ-induced diabetic rats

A significant increase (*P* < 0.01) in FBG was observed in STZ-injected rats as compared with normal control rats. Oral administration of EGA at 250, 500 and 1000 mg/kg of body weight for 30 days showed a dose-dependent and significant (*P* < 0.01) reduction in the FBG of diabetic rats as compared with diabetic control rats [Table 2]. On the 16^th^ day, the maximum reduction (34.9%) in FBG by EGA extracts was shown at the 1000 mg/kg dose as compared with the diabetic control group. Reduction in FBG was 18.95% and 21.44% for the 250 and 500 mg/kg of EGA doses, respectively. After 30 days of EGA treatment, the maximum reduction in FBG (49.39%) was shown by 1000 mg/kg as compared with the diabetic control group. The reduction in FBG was 34.24% and 42.35% for the 250 and 500 mg/kg of EGA doses, respectively. Metformin showed a 44.47% and 53.32% decrease in FBG on the 16^th^ and 31^st^ days of treatment, respectively [Table 2].

**Table 2:** Effect of the aq. extract of *Elaeocarpus ganitrus* on blood glucose levels in streptozotocin-induced diabetic rats

*Group*	*Treatment*	*Blood glucose (mg/dl)*			
		*Day 0*	*Day 15*	*Day 30*
Normal control	0.5% NaCMC	89.37 ± 3.92	93.67 ± 4.28	85.46 ± 4.81
Diabetic control	0.5% NaCMC	294.66 ± 8.07#	310.12 ± 10.85#	332.71 ± 9.22#
EGA	250 mg/kg	282.75 ± 7.47#	251.34 ± 9.09**	218.79 ± 10.08**
EGA	500 mg/kg	305.11 ± 6.96#	243.62 ± 7.61**	191.78 ± 10.75**
EGA	1000 mg/kg	286.65 ± 6.53#	201.73 ± 8.45**	168.37 ± 8.97**
Metformin	500 mg/kg	288.89 ± 5.94#	172.18 ± 7.3**	155.29 ± 9.68**

Values are expressed as mean ± SEM; *n* = 6; ^*^P < 0.05;

** *P* < 0.01 vs. diabetic control group on different days

# *P* < 0.05 vs. normal control group; one-way ANOVA followed by Dunnett’s multiple comparisons test; EGA, aqueous extract of *Elaeocarpus ganitrus*

In EGA-treated (250, 500 and 1000 mg/kg) diabetic rats, a significant decrease (*P* < 0.01) in serum triglycerides was observed after a 30-day treatment period as compared with the diabetic untreated rats. Treatment with EGA (250, 500 and 1000 mg/kg) caused a significant decrease (*P* < 0.01) in total cholesterol by 19.14%, 29.15% and 35.99%, respectively. Because of EGA treatment, the LDL cholesterol levels were also significantly decreased by 25.03%, 37.95% and 45.37%, respectively. HDL cholesterol levels were significantly (*P* < 0.01) increased by EGA treatment at 500 and 1000 mg/kg in STZ-diabetic rats by 56.39% and 60.87%. In the metformin-treated diabetic rats, there was a significant (*P* < 0.01) decrease in serum triglycerides (45.04%), total cholesterol (32.26%) and LDL (41.05%) and an increase in HDL (54.38%) as compared with diabetic control rats [Table 3].

**Table 3:** Effect of EGA on lipid profile in STZ-induced diabetes rats

*Group*	*Treatment*	*HDL-cholesterol (mg/dl)*	*Total cholesterol (mg/dl)*	*Triglycerides (mg/dl)*	*LDL cholesterol (mg/dl)*
Normal control	0.5% NaCMC	35.18 ± 197	115.87 ± 6.87	81.92 ± 4.22	64.31 ± 2.64
Diabetic control	0.5% NaCMC	21.44 ± 1.73#	216.82 ± 6.99#	174.93 ± 5.70#	160.40 ± 3.45#
EGA	250 mg/kg	27.316 ± 1.12	175.32 ± 6.24**	138.82 ± 5.03**	120.24 ± 3.52**
EGA	500 mg/kg	33.53 ± 2.66**	153.63 ± 4.36**	102.80 ± 5.62**	99.54 ± 3.03**
EGA	1000 mg/kg	34.49 ± 1.57**	138.78 ± 7.59**	83.32 ± 6.33**	87.62 ± 2.07**
Metformin	500 mg/kg	33.10 ± 1.81**	146.88 ± 5.02**	96.15 ± 3.84**	94.55 ± 0.87**

Values are expressed as mean ± SEM; *n* = 6; ^*^P < 0.05;

** *P* < 0.01 vs. diabetic control group

# *P* < 0.05 vs. normal control group; one-way ANOVA followed by Dunnett’s multiple comparisons test; EGA, aqueous extract of *Elaeocarpus ganitrus*

## DISCUSSION

In the present study, the antidiabetic effects of EGA were studied in normal and STZ-induced diabetic rats. It was interesting to note a significant hypoglycemic activity of the extract in STZ-induced diabetic rats.

STZ causes diabetes by a rapid depletion of beta-cells and, thereby, reduces insulin release and causes hyperglycemia.[11] Our study demonstrates that the EGA extract possess an antihyperglycemic activity in a dose-dependent manner on the 16^th^ and 31^st^ days of the treatment. However, the extract was not able to restore the BGL to the baseline value. This indicates that the *E. ganitrus* extract should be used with alternatives for diabetic control, like diet and hypoglycemic agents. Metformin produces hypoglycemia by an extra pancreatic mechanism.[12] The effect of EGA on BGL was lower than metformin at all the tested dose levels. Mechanism-based *in vitro* and *in vivo* studies are necessary to understand the mode of action of EGA for antihyperglycemic activity. Increased serum triglycerides and cholesterol levels in STZ-diabetic rats support previous findings.[13] EGA treatment suggests its putative role in attenuation of effects on lipid profile in diabetes.

The hypoglycemic effect of *E. ganitrus* may be attributed to alkaloids, viz. rudrakine, (-)elaeocarpine and (-)iso-elaeocarpine, flavonoids and glycosides.[1] The present investigation shows that EGA seeds have an antihyperglycemic activity in STZ-induced diabetic rats. However, further studies should be undertaken to identify the active hypoglycemic compounds and investigate the mechanism of action of the hypoglycemic activity of *E. ganitrus*.
